# Endoscopic ultrasound-guided coil embolization combined with cyanoacrylate injection into perforating vein for gastric varices

**DOI:** 10.1055/a-2719-3395

**Published:** 2025-11-06

**Authors:** Jun Li, Yingqun Zhou, Junshan Wang, Jiao Feng, Feng Liu

**Affiliations:** 1 278245 Digestive Endoscopy Center, Shanghai Tenth People’s Hospital, Tongji University School of Medicine, Shanghai, China

Gastric varices (GVs) secondary to portal hypertension pose a significant risk of life-threatening hemorrhage. While endoscopic cyanoacrylate (CYA) injection is widely used, it carries risks of embolization, rebleeding, and local complications. Endoscopic ultrasound (EUS) offers precise visualization of variceal architecture, including feeding perforating veins, enabling targeted therapy. EUS-guided interventions could minimize glue volume and reduce adverse events by directly occluding perforators.

A 62-year-old man with a history of alcohol-related cirrhosis, splenomegaly, and portal hypertension was found to have gastroesophageal varices along with portosystemic collateral vessels, as demonstrated on CT angiography ( Fig. 1 ). Endoscopy showed significantly bulged GVs in the gastric fundus measuring about 3.0 cm in diameter ( Fig. 2 ). Under general anesthesia, a linear EUS scope identified perforating veins supplying the varices ( Video 1 ). Under real-time EUS guidance, a 22-G needle punctured the perforator, deploying a 10 cm × 30 mm coil followed by 1 mL CYA injection. Real-time Doppler confirmed complete blood flow cessation ( Fig. 3 , Video 1 ). Postprocedure endoscopy showed marked varix collapse ( Fig. 4 ). An abdominal CT performed 24 hours after the procedure showed that the coils and CYA glue remained in situ ( Fig. 5 ). The patient was discharged after 48 hours without complications and remained stable at follow-ups.

**Fig. 1:**
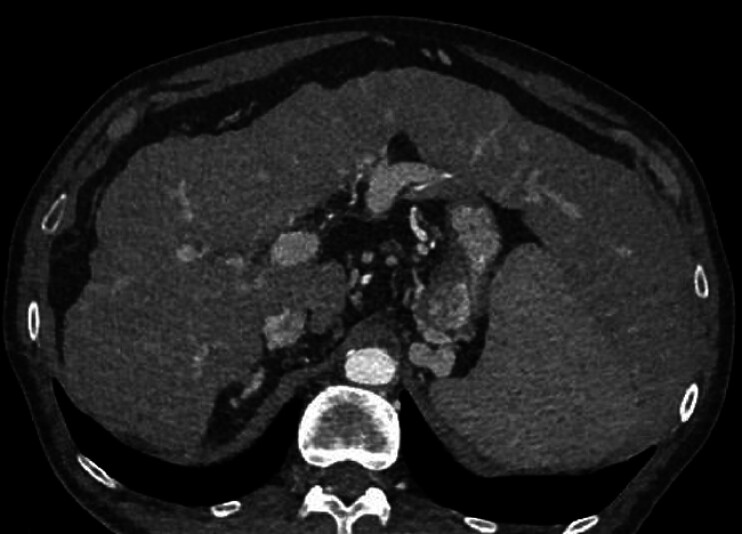
CT angiography showed cirrhosis, splenomegaly, and portal hypertension with gastroesophageal varices and portosystemic collateral vessels.

**Fig. 2:**
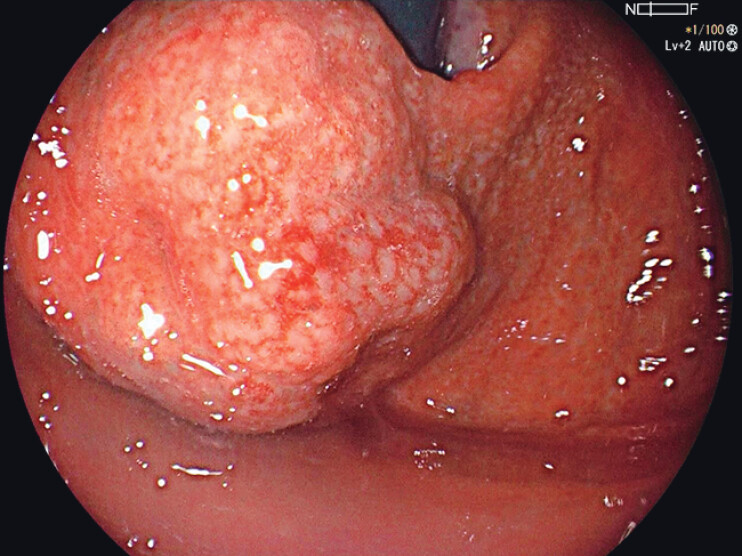
Gastroscopy showed bulged gastric varices along the greater curvature of the fundus.

EUS-guided coil embolization combined with cyanoacrylate injection into perforating vein for gastric varices.Video 1

**Fig. 3:**
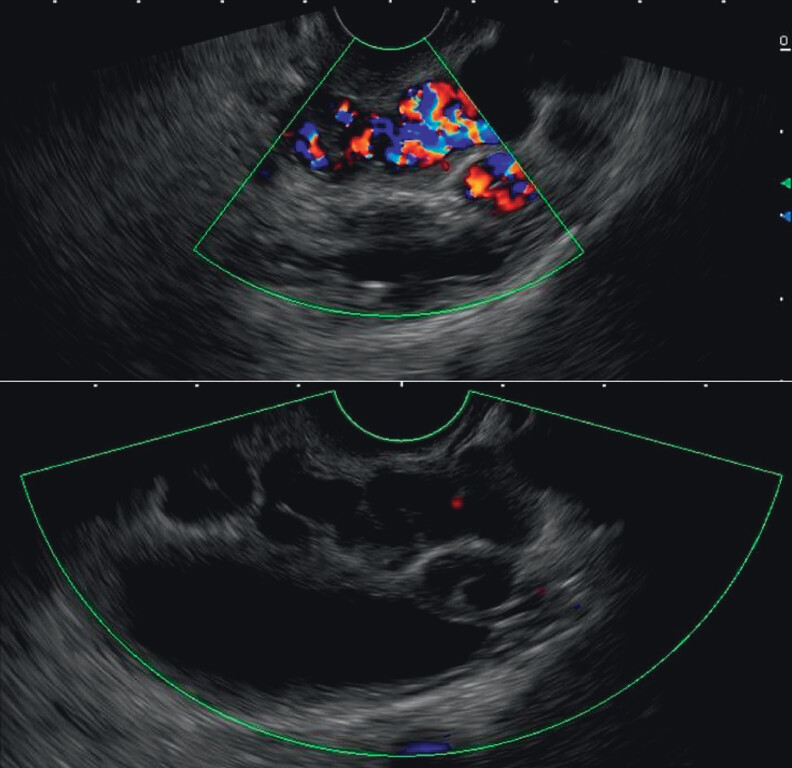
Real-time Doppler showed blood flow signals before (upper) and after (lower) the procedure.

**Fig. 4:**
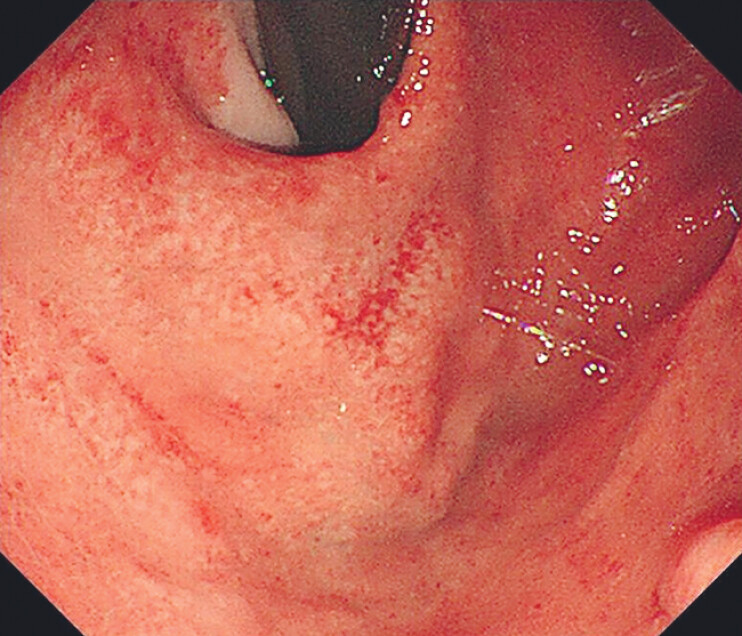
Gastroscopy after the procedure showed a significantly collapsed varix.

**Fig. 5:**
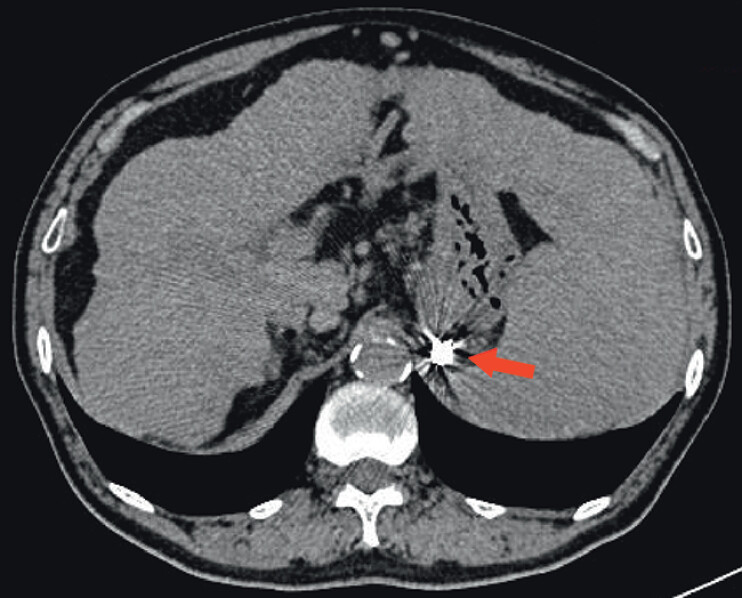
Postprocedure CT showed the coils and cyanoacrylate glue remained in situ.

This is the first reported case of EUS-guided coil embolization combined with CYA injection into a perforating vein for GV treatment. Unlike prior studies describing EUS-guided CYA alone 1 2 3 , this combined approach leverages coils as a scaffold to retain glue, minimizing migration risk and reducing required CYA volume. EUS-guided coil and CYA injection into perforating veins represents a promising, accurate technique for GV obliteration, potentially optimizing outcomes by enhancing precision and reducing glue-related risks.

Endoscopy_UCTN_Code_TTT_1AS_2AB Endoscopy_UCTN_Code_TTT_1AO_2AD

## Notes

Endoscopy E-Videos https://eref.thieme.de/e-videos
*E-Videos* is an open access online section of the journal *Endoscopy* , reporting on interesting cases and new techniques in gastroenterological endoscopy. All papers include a high-quality video and are published with a Creative Commons CC-BY license. Endoscopy E-Videos qualify for HINARI discounts and waivers and eligibility is automatically checked during the submission process. We grant 100% waivers to articles whose corresponding authors are based in Group A countries and 50% waivers to those who are based in Group B countries as classified by Research4Life (see: https://www.research4life.org/access/eligibility/ ). This section has its own submission website at https://mc.manuscriptcentral.com/e-videos .
